# Activity of tea tree oil and nerolidol alone or in combination against *Pediculus capitis* (head lice) and its eggs

**DOI:** 10.1007/s00436-012-3045-0

**Published:** 2012-07-31

**Authors:** Emanuela Di Campli, Soraya Di Bartolomeo, Patricia Delli Pizzi, Mara Di Giulio, Rossella Grande, Antonia Nostro, Luigina Cellini

**Affiliations:** 1 Department of Drug Sciences, University of “G. d’Annunzio”, Chieti–Pescara, Italy; 2 Union Health S.r.l, S. Giovanni Teatino, Chieti Italy; 3 Pharmaco-Biological Department, Faculty of Pharmacy, University of Messina, Messina, Italy

## Abstract

Head lice infestation is an emerging social problem in undeveloped and developed countries. Because of louse resistance increasing, several long-used insecticidal compounds have lost their efficacy, and alternatives, such as essential oils, have been proposed to treat this parasitic infestation. The present study investigated the efficacy of two natural substances: tea tree (*Melaleuca alternifolia*) oil and nerolidol (3,7,11-trimethyl-1,6,10-dodecatrien-3-ol) against lice and its eggs. Products were used alone and in combination (ratio 1:1 and 1:2) from 8 % dilution. The in vitro effect of natural substances at different concentrations were evaluated against 69 head lice (adults and nymphs) and 187 louse eggs collected from school children in Chieti–Pescara (Central Italy) over a 6-month period. The lice mortality was evaluated for 24 h by a stereo light microscope. The ovicidal activity was monitored by microscopic inspections for 15 days. Tea tree oil was more effective than nerolidol against head lice with 100 % mortality at 30 min and 1 % concentration. On the contrary, nerolidol expressed a more pronounced ovicidal activity inducing the failure of 50 % of the eggs to hatch at 1 % concentration after 4 days; the same effect was achieved by using a twice concentration of tea tree oil. The association of the two substances both in ratios 1:1 and 1:2 combined efficaciously their insecticidal and ovicidal effect; in particular, the ratio 1:2 (tea tree oil 0.5 % plus nerolidol 1 %) acted producing both the death of all head lice at 30 min and the abortive effect of louse eggs after 5 days. These results offer new potential application of natural compounds and display a promising scenario in the treatment of pediculosis resistant cases. The development of novel pediculicides containing essential oils could be, in fact, an important tool to control the parasitic infestation.

**Electronic supplementary material:**

The online version of this article (doi:10.1007/s00436-012-3045-0) contains supplementary material, which is available to authorized users.

## Introduction

Head lice infestation is caused by *Pediculus humanus capitis* De Geer, belonging to the family Pediculocidae, permanent and strictly obligatory ectoparasite species-specific: it completes the entire life cycle on the scalp of man. The head louse is a hematophagous that survives by sucking the blood, several times a day (every 2–3 h).The male *P. humanus capitis* (2–3 mm) is smaller than the female (3–4 mm); the female is the most important vector of infection, since it lives 30 days after fertilization and lays 8–10 eggs per day for a total of 50–300 eggs during its lifetime. From the eggs (nits), nymphs will hatch after 8–12 days and become mature in another 8 days. One to two eggs are laid on the hair, at few millimeters from the follicle, grayish white, shiny surface, and adhering to the hair with a cementing substance. After 6–10 days, the larvae from the egg slip out of the site of the operculum (Burgess 2004; Chosidow 2000; Ko and Elston 2004; CDC 2005).

The transmission may be direct, from one head to another when they are very close, or indirect, through clothing (hats, caps, headbands muffs, shawls, scarves, and jackets), the common use of hair brushes combs, through the bedding, the backs of upholstered chairs, blankets, stuffed animals, and the common use of wardrobes (Leung et al. 2005).

The infestation may be asymptomatic or symptomatic; in symptomatic cases, the itching is found in a highly variable percentage of patients (Chosidow 2000). The itching can be caused both by the bite of lice on the skin and the irritative-allergic reaction caused by the deposition of saliva on the scalp. The symptoms occur when the infestation is already old (Chosidow 2000; Flinders and De Schweinitz 2004).

Many parents overlook this problem or are not sufficiently informed. Although the infestation is characterized by a low degree of morbidity, because of its high prevalence, not to be excluded the psycho-social consequences, with exclusion of affected patients, this condition is considered socially inappropriate (Mumcuoglu 1999). Head lice infestation is spread throughout the world and represents an important problem from the standpoint of social health. In Italy, head lice infestation appears among children in nursery and primary schools, especially during the cold months (autumn–winter) and also in summer (swimming pool and colonies) (Canyon and Speare 2007a; Sidoti et al. 2009).

Several topical head lice treatments based on insecticidal chemicals failed to obtain a lice control for their misuse or extensive use favoring the emergence of resistance, especially in developed countries (Hunter et al. 2003; Roberts and Burgess 2005; Mumcuoglu 2006; Priestley et al. 2006; Burgess 2009a; 2009b; Burgess and Burgess 2011). As a consequence, the presence of lice infestation is an increasing problem (Lee et al. 2000; Roberts and Burgess 2005), and the effectiveness of alternative compounds should be considered to evaluate the development of novel pediculicides.

Plant-based compounds such as the flowers bud extract of *Syzygium aromaticum*, *Melia azedarach*, lavender oil, eucalyptus oil, lemon tea tree oil, thymol, and geraniol have been taken into account for their activity against both insects and their eggs and could represent an interesting approach to limit the emergence and the spread of the parasitic infestation (Heukelbach et al. 2006; Abdel-Ghaffar et al. 2007; Priestley et al. 2006; Carpinella et al. 2007; Bagavan et al. 2011; Barker and Altman 2011; Mehlhorn et al. 2011; Abdel-Ghaffar et al. 2012).

Tea tree oil is a compound derived from the Australian native plant *Melaleuca alternifolia*. It has well-established quality control procedures, and its composition has specified under International Organization for Standardization standard 4730 (oil of Melaleucaterpinen-4-ol type) (ISO 1996). Tea tree oil is reported to have a wide variety of biological properties, including antimicrobial, anti-inflammatory, anticancer, and insecticidal activities (Gould 1997) with interesting applicative prospects. The insecticidal properties (Williamson et al. 2007; Heukelbach et al. 2008) could be useful in the treatment of larvae in strikes, the repellent effects (Canyon and Speare 2007b; Eamsobhana et al. 2009; Maguranyi et al. 2009) could protect against new strikes or restrikes, and the antimicrobial and anti-inflammatory activities (Carson et al. 2006) can favor wound healing (Woollard et al. 2007).

Nerolidol or 3,7,11-trimethyl–1,6,10-dodecatrien-3-ol is a predominant sesquiterpene, which not only occurs in *Baccharis dracunculifolia* essential oil but is also found in many essential oils (Klopell et al. 2007). Nerolidol is a fragrance ingredient and has been studied as a topical skin penetration enhancer (Lapczynski et al. 2008; Williams and Barry 2004). Moreover, it has been documented to have significant antibacterial (Brehm-Stecher and Johnson 2003; Inoue et al. 2004), antifungal (Lee et al. 2007), antimalarial (Lopes et al. 1999), antileishmanial (Arruda et al. 2005), and antiulcer activities (Klopell et al. 2007). In particular, Priestley et al. (2006), found a significant lethality against louse eggs at a concentration of 1 %.

The aim of this study was to evaluate the activity of tea tree oil and nerolidol against eggs, nymphs and adults of *Pediculus capitis* used alone and in combination, in ratios 1:1 and 1:2.

## Materials and methods

### Head lice and louse eggs collection

Adults, nymphs, and eggs of *P. capitis* were collected from the head of children 6–13 years old, who attended primary/elementary schools in Chieti–Pescara (Central Italy) over a 6-month period.

**Fig. 1:**
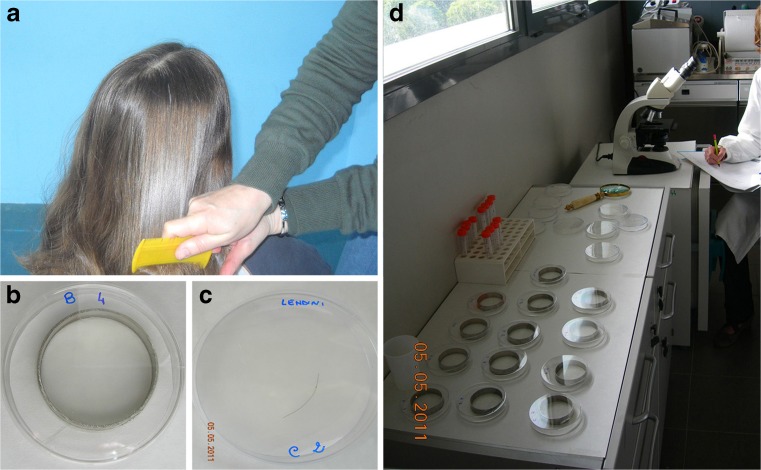
Head lice and louse eggs detection. (**a**) Collection of lice by combing from infested head. (**b**) Basket for lice. (**c**) Viable louse egg attached to the hair. (**d**) Lice and louse eggs treated for experiments

The children were not previously treated with antilice products for at least 1 month, and the head lice were collected using a fine-toothed antilouse metal comb (Fig. 1a) and transported to the laboratory in glass jars with screw caps, with the approval of the children guardians. We conducted a total of seven experiments.

A total of 69 head lice (21 adults and 48 nymphs) were analyzed and considered indistinctly. Lice, processed within 2 h after collection, were placed into 6-cm diameter stainless steel baskets with nylon net bottoms into separate glass Petri dishes lined with Whatman no. 1 filter paper (7.0 cm in diameter) on the bottom (Fig. 1b) and used for the experiments.

Louse eggs, attached <1 cm from the scalp, were collected by cutting the hair with hairdresser scissors and put in glass jar with screw caps in a polystyrene container to minimize temperature variation. The samples were taken to the laboratory and processed within 2 h after collection. One hundred eighty-seven louse eggs were selected among the 250 or more microscopically observed due to their homogenous aspects, with closed operculum and in the same phase of embryonic development (Mougabure-Cueto et al. 2006). These, then were placed in separate glass Petri dishes lined with Whatman no. 1 filter paper (7.0 cm in diameter) on the bottom (Fig. 1c) and used for the experiments.

### Products

**Fig. 2:**
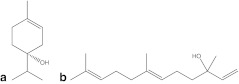
Chemical structure of terpinen-4-ol (**a**), the main component of tea tree oil, and nerolidol (**b**)

Tea tree oil (oil of melaleucaterpinen-4-ol type, Fig. 2a) and nerolidol (3,7,11-Trimethyl-1,6,10-dodecatrien-3-ol, Fig. 2b) were purchased from A.C.E.F. SpA (Fiorenzuola D’adda, Piacenza, Italy) and Moellhausen SpA (Vimercate, Milano, Italy), respectively. Both products were dissolved in ethylexyl stearate to obtain the following twofold solutions: tea tree oil (A), 8, 4, 2, and 1 %; nerolidol (B), 8, 4, 2, and 1 %. The essential oils were also combined in ratios 1:1 and 1:2, obtaining the following combinations:

- Tea tree oil plus nerolidol, ratio 1:1 (C): C1 = A4 % plus B4 %, C2 = A2 % plus B2 %, C3 = A1 % plus B1 %, C4 = A0.5 % plus B0.5 %
- Tea tree oil plus nerolidol, ratio 1:2 (D): D1 = A4 % plus B8 %, D2 = A2 % plus B4 %, D3 = A1 % plus B2 %, D4 = A0.5 % plus B1 %.

### Bioassay

#### Pediculicidal activity

For testing pediculicidal activity, the baskets containing adults and nymphs (detected together) of lice, distributed in Petri dishes lined with Wathman no.1 paper filter on the bottom (Fig. 1d), were treated with 600 μl of each product (A–D) at the different concentrations and in the two tested combinations for 15 min and at 65 ± 5 % humidity in darkness chamber and incubated at 35 ± 2 °C (WHO 1981). Then, to simulate the treatment of an infested host, the baskets were taken out, and the lice were washed with top water until they were completely free from residual products and placed in Petri dishes with untreated filter papers and incubated at 35 ± 2 °C and at 65 ± 5 % humidity in darkness. Two control tests were performed: one with lice placed on unimpregnated filter paper, and another with lice exposed to solvent ethylexyl stearate impregnated filter paper dried for 5 min under fume cupboard. The plates with adults and nymphs of lice were observed by stereomicroscope at 10, 15, 20, 30, 60, 120, 240 min and 24 h. Depending on the insects recovering, each basket for each experiment could contain one, two, or three lice.

Criteria of death of head louse were extremely strict and were defined as absence of movement of limbs and gut, with or without stimulation using forceps. Experiments were repeated, at least, twice.

#### Ovicidal activity

For testing ovicidal activity, all the viable selected louse eggs, distributed in Petri dishes with Wathman no.1 paper filter on the bottom (Fig. 1d), were embedded with 600 μl of each product (A–D) at the different concentrations and in the two tested combinations. After the exposure, the filter papers were dried for 5 min under a fume cupboard. Two control tests were performed: one with louse eggs placed on unimpregnated filter paper, and another with louse eggs exposed to solvent ethylexil stearate impregnated filter paper dried for 5 min under a fume cupboard; treated samples and controls were incubated at 37 ± 2 °C and 65 ± 5 % humidity chamber in darkness for 15 days. Depending on the louse eggs recovering, each Petri dish for each experiment could contain four or five eggs.

The louse eggs hatching were monitored daily under microscopic inspection. Mortality data of treated eggs were recorded 5 days after the hatching of controls. Louse eggs with closed operculum and nymphs inside were the criterion for embryo mortality (abortive eggs) (Mougabure-Cueto et al. 2006). Experiments were repeated, at least, twice.

## Results

**Fig. 3:**
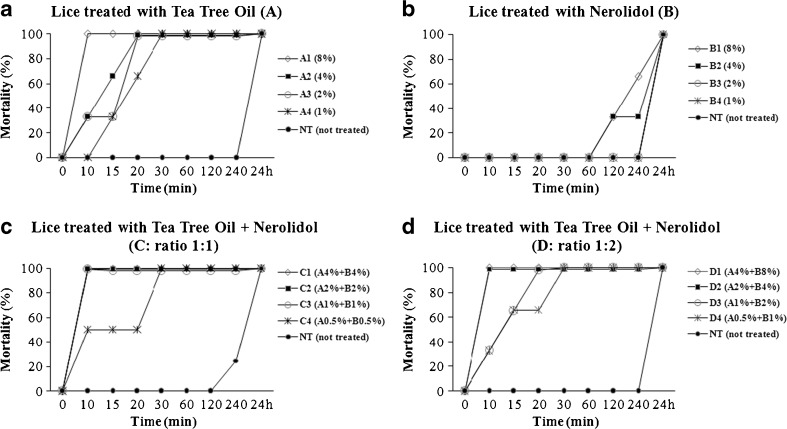
Mortality (%) of head lice (adults and nymphs) treated with tea tree oil (**a**), nerolidol (**b**), and their combination in ratio of 1:1 (**c**) and 1:2 (**d**) at different concentrations (see “Materials and methods”) and control groups, in time. The total number of lice ranged from 2 to 10, for each product and for each concentration

The results obtained by treating 69 head lice, adults and nymphs, with tea tree oil (A), nerolid (B) and their combinations in ratios 1:1 (C) and 1:2 (D) at different concentrations are shown in Fig. 3.

The percentage of lice mortality, detected in 24 h, was expressed using highly strict criteria for mortality (no external or internal vital signs). Lice treated with tea tree oil at 1 % of concentration (A4), caused 100 % mortality after 30 min of washing (Fig. 3a). The treatment with different concentrations of nerolidol was less effective than tea tree oil with 33 % mortality reached at 2 % (B3) after 120 min of washing (Fig. 3b).

**Fig. 4:**
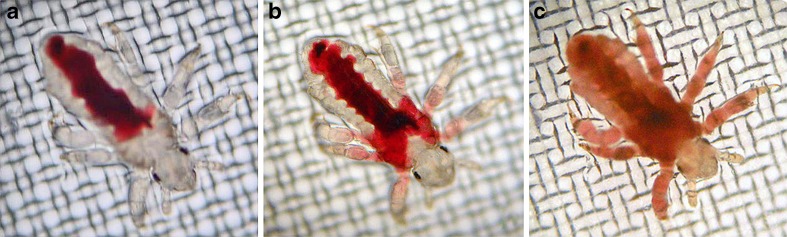
Nymph of louse dead after 20 min of treatment with tea tree oil at 1 % concentration (A4) showing the gut rupture (**a**), with seepage into the thorax (**b**) and limbs (**c**), which appeared after 30 and 60 min, respectively

Combining the two essential oils, no vital signs were recorded in the 100 % of lice after 30 min of washing at C4 condition (A0.5 % plus B0.5 %); the same efficacy was achieved after 10 min of washing at C3 combination with essential oils at 1 % each (Fig. 3c). The mixing of tea tree oil and nerolidol in the ratio 1:2 produced 100 % mortality after 20 min of washing at D3 condition (A1 % plus B2 %) (Fig. 3d). The evaluation of vital signs was recorded both in nymphs and adult lice resulting in a more resistance to treatments of nymphs than adult lice, in each examined group, with persistent internal movements (Video 1) also in presence of dead adult insects (Video 2) until the total mortality occurred. Interestingly, dead nymphs were mainly characterized by the gut rupture with seepage into the thorax and limbs (Fig. 4).

All the head lice in the negative control group survived during the observation time for 240 min except for C condition (Fig. 3c) with 25 % mortality; the same survival values were recorded, in each examined group, also after 6 h of observation (not shown); after 24 h, all insects were dead.

**Fig. 5:**
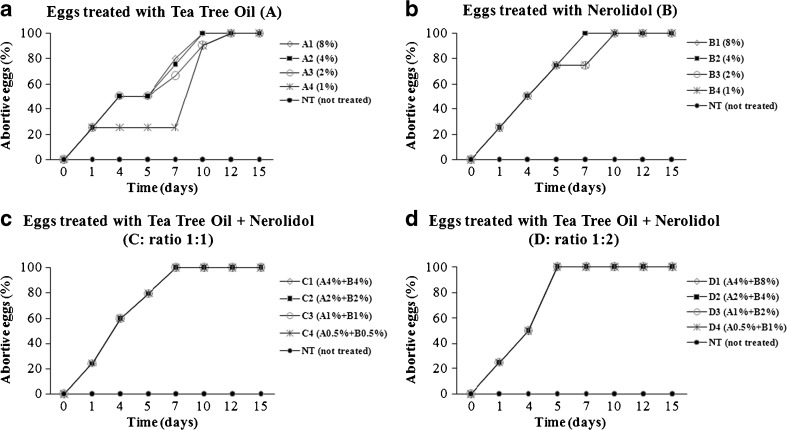
Abortive (%) louse eggs treated with tea tree oil (**a**), nerolidol (**b**), and their combination in ratio 1:1 (**c**) and 1:2 (**d**) at different concentrations (see “Materials and methods”) and control groups, in time. The total number of louse eggs ranged from 8 to 12, for each product and for each concentration

The ovicidal activity of tea tree oil (A), nerolidol (B), and their combinations in ratios 1:1 (C) and 1:2 (D) is summarized in Fig. 5. Few detectable modifications in the development of larvae inside the eggs were recorded with tea tree oil at lower concentrations; the 50 % of abortive eggs at 2 % concentration was reached after 4 days of observation (Fig. 5a). The treatment with nerolidol was more effective against louse eggs in respect to tea tree oil; in fact, after the same time of observation (4 days), the 50 % of ovicidal effect was reached at 1 % concentration, and, after 7 days, all louse eggs were abortive (Fig. 5b).

**Fig. 6:**
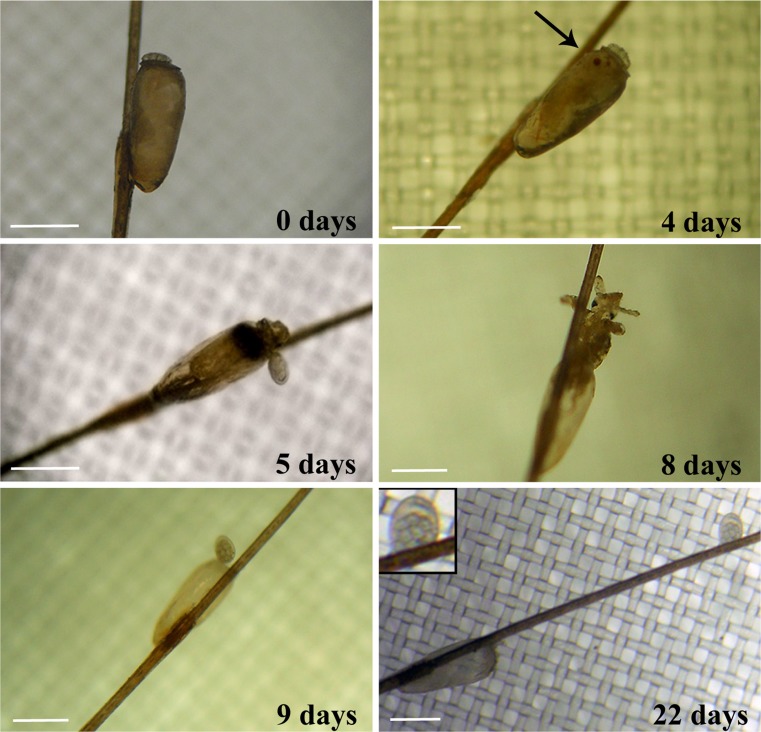
Phases of hatching of louse egg, in time. Eye spot (*arrow*). Operculum with aeropyles (*inset*). Original magnification ×100. Scale bar = 500 μm

Regarding the essential oil combinations, the mixture C4 (A0.5 % plus B0.5 %) was effective in the 80 % of treated louse eggs after 5 days (Fig. 5c), whereas the mixture D4 (A0.5 % plus B1 %) induced, after the same period of time, the full ovicidal effect (Fig. 5d). Louse eggs in the controls hatched after 5–8 days (Fig. 6).

## Discussions

The present study aimed to evaluate the efficacy of two natural products, tea tree oil and nerolidol, at various concentrations, used alone or combined in ratios 1:1 and 1:2, against lice and their eggs. The in vitro tests displayed a significant efficacy at lower concentrations. In particular, tea tree oil needed the shortest time (30 min) until all lice were killed at 1 % concentration, whereas the most effective oil for killing louse eggs appeared to be nerolidol that provided the 75 % abortive eggs 5 days after treatment. This ovicidal effect was also detected by Priestley et al. (2006), who attributed the specific action of nerolidol against louse eggs to its bioavailability favoring the penetration through the highly hydrophobic cuticle.

When the analyzed products were mixed, the pediculicidal effect of tea tree oil and the prevalent ovicidal activity of nerolidol were usefully displayed; in particular, in ratio 1:2, at less concentration (D4), the total lice killing was obtained within 30 min and the complete inhibition of nymphs emergence happened at 5 days, whereas the association at D3 concentration (A1 % + B2 %) produced a 100 % mortality of adult insects and nymphs after 20 min of exposure.

The considerations agreed by other authors (Carpinella et al. 2007; Priesley et al. 2006) and the results obtained from this study suggest that the combination investigated can improve the insecticidal activity, in particular, when lice are resistant. Other studies (Bagavan et al. 2011; Mehlhorn et al. 2011; Abdel-Ghaffar et al. 2012) analyzed the effect of natural products against both motile lice and eggs. In particular, Abdel-Ghaffar et al. (2012) propose an antilice shampoo based on a new seed extract able to be efficacious after one treatment. The hexane flower bud extract of *S. aromaticum* (*Myrtaceae*) was evaluated by Bagavan et al. (2011), who found a major effect in vapor phase. These studies underline that natural products may provide good antilice activity and can offer a valid alternative to conventional insecticides. In our study, the investigated substances, in the ratio of 1:2, make up the formulation of the antihead lice natural product NOPID®, used in Italy and indicated for the treatment of head lice and its eggs.

The results obtained from this study presented a promising scenario for using combinations of tea tree oil and nerolidol as effective alternative for treating pediculosis.

Essential oils are potential natural products for lice control, promoting selective effects against resistant insects, and may prevent the rapid development of resistance, in particular in developed countries (Mumcuoglu et al. 2002). In Italy, very few studies were carried out (Sidoti et al. 2009), and misdiagnosis, overuse of pediculicides, and lack of information contribute both to the lice resistance and the difficulty of survey. Moreover, studies of pediculicidal and ovicidal effectiveness of new products should be performed with lice collected in the country to obtain real efficacious treatments.

Our analysis provides efficacy against lice and their eggs encouraging for novel therapy schemes including natural compounds as alternative approaches to cure *P. capitis* infestations.

Moreover, in this study, we avoid misidentifying mortality following stringent criteria for the definition of mortality of lice and louse eggs. As observed by Burgess (2009a) using dimeticone 4 %, in our study, we found gut disruption with evidences of seepage of the thorax and the limbs in head lice.

As suggested by Yang et al. (2005), the mode of delivery of the essential oil and compounds was likely by vapor action via respiratory system blocking. The essential oils are complex mixtures of many different components with various degrees of lipophilicity, relative hydrophilicity, and volatility at room temperature.

The terpenoid components of essential oils as tea tree oil can exert their action in a mechanical way.

Because of the low molecular weight, such compounds can pass through the cuticle of louse up to the trachea causing the death of head lice by suffocation. The efficacy of essential oils can be attributable to the combined effect of direct deposition of essential oils on cuticle together with an indirect effect via adsorption of the vapors. Then, to potentiate this effect, it will be useful to apply oils, cover hair with a shower cap, and let stand overnight with the aim to suffocate the remaining live lice (nymphs).

The results of this study offer new potential application of natural compounds. The development of novel pediculicides containing essential oils could be, in fact, an important tool to control the parasitic infestation.

Education of parents and teachers about louse biology, epidemiology, control, and an effective prevention is of paramount importance to manage lice infestation. In fact, the increasing knowledge of the pediculosis, overcoming prejudice, should favor a rapid alert of health service, providing suitable treatment.

## Electronic supplementary material

Nymph showing persistent internal movements of gut (Video 1) and dead adult louse and nymph showing persistent movements of limbs (Video 2) after 30 min of treatment at D4 condition (tea tree oil 0.5 % plus nerolidol 1 %).

ESM 1 (MPG 444 kb)

ESM 2 (MPG 756 kb)
